# The cheetah optimizer: a nature-inspired metaheuristic algorithm for large-scale optimization problems

**DOI:** 10.1038/s41598-022-14338-z

**Published:** 2022-06-29

**Authors:** Mohammad Amin Akbari, Mohsen Zare, Rasoul Azizipanah-abarghooee, Seyedali Mirjalili, Mohamed Deriche

**Affiliations:** 1 Artificial Intelligence Research Centre, Ajman University, Ajman, United Arab Emirates; 2 Department of Electrical Engineering, Faculty of Engineering, Jahrom University, Jahrom, Fars, Iran; 3 National Grid ESO, Warwick, CV346DA UK; 4 Centre for Artificial Intelligence Research and Optimisation, Torrens University Australia, Brisbane, Australia; 5 Yonsei Frontier Lab, Yonsei University, Seoul, South Korea

**Keywords:** Engineering, Mathematics and computing

## Abstract

Motivated by the hunting strategies of cheetahs, this paper proposes a nature-inspired algorithm called the cheetah optimizer (CO). Cheetahs generally utilize three main strategies for hunting prey, i.e., searching, sitting-and-waiting, and attacking. These strategies are adopted in this work. Additionally, the leave the pray and go back home strategy is also incorporated in the hunting process to improve the proposed framework's population diversification, convergence performance, and robustness. We perform intensive testing over 14 shifted-rotated CEC-2005 benchmark functions to evaluate the performance of the proposed CO in comparison to state-of-the-art algorithms. Moreover, to test the power of the proposed CO algorithm over large-scale optimization problems, the CEC2010 and the CEC2013 benchmarks are considered. The proposed algorithm is also tested in solving one of the well-known and complex engineering problems, i.e., the economic load dispatch problem. For all considered problems, the results are shown to outperform those obtained using other conventional and improved algorithms. The simulation results demonstrate that the CO algorithm can successfully solve large-scale and challenging optimization problems and offers a significant advantage over different standards and improved and hybrid existing algorithms. Note that the source code of the CO algorithm is publicly available at https://www.optim-app.com/projects/co.

## Introduction

### Background

Recently, solving optimization problems have become a challenging and exciting topic in most research area. Decision-making problems that are overgrowing can be defined as optimization problems. An optimization problem includes one or more objective functions, decision variables, and constraints to be minimized or maximized^1^. Many deterministic approaches, such as linear programming, Newton methods, quadratic programming, dynamic programming, simplex method, gradient method, etc., are the well-known classical methods to solve optimization problems^2,3^. These algorithms robustly result in the same solution for a given optimization problem with an identical initial starting point. Although such techniques can find optimal solutions in a reasonable time, they need the objective function and constraints to be convex and derivable^4^. These cause the deterministic algorithms to fall into locally optimal solutions, which is the main shortcoming of such methods in solving real-world problems. This defect becomes more prominent as the dimension of the problem increases. Therefore, stochastic methods for dealing with it have been developed^5–7^. These algorithms intrinsically neglect the characteristics of the objective functions and constraints, so they treat the problem as a black box. Another advantage of the most metaheuristic algorithms is their simplicity.

How metaheuristics solve a problem is similar. Evolve a set of random solutions in an iterative procedure and keep a good balance between exploration and exploitation phases^8^. To this end, the search process is divided into two stages exploration and exploitation^9^. The former assists an algorithm to search globally, and the latter helps it locally search around the probable region obtained by the former phase^10^. The common feature in metaheuristic algorithms is their randomness which can help them to avoid local solutions. However, these features cause metaheuristic algorithms not to guarantee the global solution. Furthermore, they may result in different solutions in each run^11^.

According to the No Free Lunch (NFL) theorem^12^, there is no unique algorithm to solve all optimization problems effectively. This theorem motivates researchers to introduce new algorithms to be applied in various fields of study^13^. Many recently developed nature-inspired metaheuristic algorithms evidenced this theorem and encouraged us to introduce a novel nature-inspired algorithm based on cheetahs' hunting strategies.

### Literature review

Metaheuristic algorithms can be categorized into two main classes, single-solution-based and multiple-solution-based (or population-based). The most popular single-solution-based metaheuristic algorithm is simulated annealing (SA)^14^. This algorithm's process starts with a random candidate solution (a population) and then moves and improves it in the promising search space in an iterative manner to find the superior solution. However, multiple-solution-based algorithms implement more than one random candidate solution to enhance the speed and the chance to avoid local optima entrapment.

Population-based metaheuristic algorithms can be classified into evolutionary, physics, chemistry, and swarm-based algorithms^15^. Most popular evolutionary-based metaheuristic algorithms are genetic algorithm (GA)^16^, genetic programming (GP)^17^, evolutionary programming (EP)^18^, evolutionary strategy (ES)^19^, biogeography-based optimizer (BBO)^20^ and differential evolution (DE)^21^. The basis of population improvement and movement in these algorithms is derived from the concept of evolution in nature.

Physics-based metaheuristic algorithms are another type of population-based optimization algorithm. The improvement and movement of the population through search space in these algorithms is made by directly deploying the known laws of physics. These rules include the laws of mechanics, relativity, gravity, electrodynamics, electromagnetism, optics, etc.^15^. The most famous of these algorithms only cover those which have at least 100 citations, as measured by Google Scholar (collected in March 2021) are, respectively: gravitational search algorithm (GSA)^22^, big bang-big crunch (BB-BC)^23^, charged system search (CSS)^24^, the electromagnetism-like mechanism (EM)^25^, water cycle algorithm (WCA)^26^, extremal optimization (EO)^27^, ray optimization (RO)^28^, central force optimization (CFO)^29^, intelligent water drops (IWD)^30^, chaos optimization algorithm (COA)^31^, galaxy-based search algorithm (GBSA)^32^ and river formation dynamics algorithm (RFDA)^33^.

Another class of population-based metaheuristic algorithms is chemistry-based algorithms. These algorithms are created based on molecules' chemical reactions and features. The most popular chemistry-based algorithms are artificial chemical reaction optimization algorithm (ACROA)^34^, gases Brownian motion optimization (GBMO)^35^ and artificial chemical process (ACP)^36^.

The most popular class of population-based metaheuristic algorithms for researchers is swarm-based algorithms. This type of algorithm is a model of the behavior and social intelligence of a group of living things, such as birds, ants, swarms, schools and so on^15^. Some of the most popular algorithms in this category are particle swarm optimization (PSO) in 1995^37^, ant colony algorithms (ACO) in 1991^38^, artificial bee colony (ABC) in 2007^39^, Cuckoo Search (CS) in 2009^40^, grey wolf optimizer (GWO) in 2014^41^, firefly algorithm (FA) in 2009^42^, bacterial foraging algorithm (BFA) in 2002^43^, whale optimization algorithm (WOA) in 2016^44^, bat algorithm (BA) in 2010^45^, Shuffled frog leaping algorithm (SFLA) in 2003^46^, bees algorithm (BA) in 2006^47^, moth-flame optimization (MFO) in 2015^48^, krill herd (KH) in 2012^49^, ant lion optimizer (ALO) in 2015^50^, fruit fly optimization algorithm (FOA) in 2012^51^, and glowworm swarm optimization (GSO) in 2009^52^.

### Motivation

Some swarm intelligence algorithms are based on animals' hunting and foraging behaviors in nature. Some hunters can hunt the prey individually or in a herd with some numbers, and other members may not participate in the hunting process. Furthermore, in some cases, a small number of hunters can cover a large hunting area. These special features of the cheetah for hunting motivated us to study its behavior more carefully and base it on the development of an optimization algorithm. The hunting processes are modeled in two simple sit and wait beside the attacking mode strategies. Indeed, despite other methods which use some complicated equations in the evolution process, the cheetah optimizer (CO) employs some simple techniques, while the hunting strategies help increase the algorithm's effectiveness. Sitting and waiting to make the prey available, back to home in case of unsuccessful hunting process, return to last successful hunting if the prey not found for sometimes. These are the main strategies in CO. The algorithm performance confirms that the hunting process's characteristics have been modeled in the proposed CO.

### Contributions

The main contributions of this paper are listed as follows:

1. A new population-based metaheuristic called CO algorithm is investigated, formulated, and tested on different benchmark functions.
2. Since the hunting processes of cheetahs, i.e., search, sit-and-wait, attack, and leave the prey and go back home, are modeled wholly and simply; thus, the number of initial populations are decreased dramatically in small to large scale optimization problems.
3. The proposed CO method needs a small number of equations while the hunting strategies try to model the hunting process. These strategies create a suitable trade-off between the exploration and exploitation searches and prevent premature convergence in the different optimization problems. Therefore, the concepts of proposed strategies can be used to enhance the performance of other metaheuristic algorithms effectively.

It should be noted that there are existing algorithms in the literature inspired by cheetah^53–55^.﻿ In^53^, it has been tried to use the cheetahs' feature in the chasing mode as an optimization algorithm, but the model is not well shown conceptually and mathematically. In^54^, an algorithm based on the hunting behavior of cheetahs was introduced. The model has been established based on GWO and modified to adapt to the group hunting behavior of cheetahs, such as leadership hierarchy and communication between teammates during the hunting. However, these algorithms have not been able to make good use of all the features and strategies of cheetahs during hunting and model them mathematically. This article tries to cover these shortcomings well to present a more realistic behavior of cheetahs in creating a robust meta-heuristic algorithm.

The rest of this paper is organized as follows. The inspiration for the suggested method is briefly addressed in “Inspiration” section. “Mathematical model and algorithm” section presents the proposed CO algorithm's mathematical model. “Simulation results” section presents experimental findings and discussions for various benchmark test functions and economic load dispatch (ELD) problems. Finally, concluding remarks and future work are summarized in “Conclusion” section.

## Inspiration

Cheetah (Acinonyx jubatus) is the primary cat breed and fastest land animal living in the central areas of Iran and Africa^56^. The cheetah's speed can reach over 120 km per hour. The cheetahs' agility and speed are their physical characteristics like a long tail, long and thin legs, lightweight and flexible spine. Cheetahs are quick animals capable of stealthy movement, fast returning during predation, and specific spotted coats; however, these visual predators cannot maintain their high-speed action for a long time. Therefore, the chasing must be less than half of a minute^57^.

Moreover, their speed significantly decreases from 93 km/h or 58 mph to 23 km/h 14 mph only in three strides after catching the prey. Due to the mentioned limitation of cheetahs in maintaining their speed, they precisely observe the environment after staying on small branches or hills to identify their prey. Furthermore, these big cats can effortlessly blend into the high and dry grass due to their specific coats^58^.

These predators usually hunt gazelles, specifically Thomson's gazelles, impalas, antelopes, hares, birds, rodents, and calves of more fabulous herd animals. First, they move slowly toward the prey with a crouched posture to be hidden and reach the minimum distance, stopping hidden and waiting for the prey to approach the predator. This is because they stop hunting if the prey observes the predator. The mentioned minimum distance is almost 60–70 m or 200–230 ft; however, it is determined to be 200 m or 660 ft if they cannot stay hidden appropriately. Specifically, the pursuit duration is 60 s with a mean distance of 173 m or 568 ft to 559 m or 1834 ft. Then, the prey's balance is lost after their rump is beaten with the cheetah's forepaw, and finally, the predator brings down the prey using too much force and turns it, which makes the prey try to escape^59^. Cheetahs' muscular tails' back and forth movement also helps them achieve sharp turns^60^. Generally, hunting the animals that move far from their herds or have less caution is much easier^61,62^. It should be noted that there are various determinants associated with predation, including maturity, gender, the number of predators, and the carelessness of prey. Also, coalitions or mothers with cubs tend to hunt more giant animals successfully.

According to the biological investigations, it has been found that cheetahs have remarkable spinal flexibility and long tails that lead to their physical balance. Moreover, they have collarbone-separated shoulder blades that facilitate the movement of the shoulders. All the characteristics mentioned earlier make these big cats considered remarkable predators; however, not all the predations are successful.

## Mathematical model and algorithm

**Figure 1:**
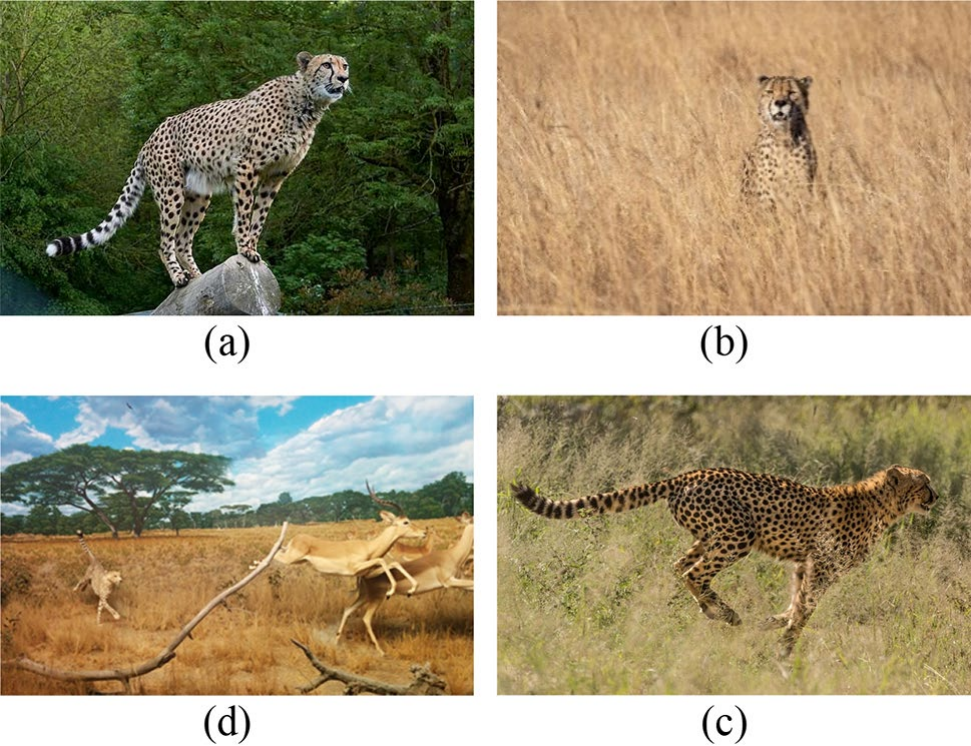
Hunting behavior of cheetahs: (**a**) searching for prey (scanning mode), (**b**) sitting-and-waiting (hiding), (**c**) rushing and (**d**) capturing.

When a cheetah is patrolling or scanning its surroundings, it is possible to detect prey. Seeing the prey, the cheetah may sit in its place and wait until the prey gets closer to it and then starts the attack. The attack mode includes rushing and capturing phases. The cheetah may give up the hunting for several reasons, such as its energy limits, fast prey fleeing, etc. Then, they may go back home to rest and start new hunting. By assessing the prey, his/her condition, area and distance to the prey, the cheetah may choose one of these strategies, as depicted in Fig. 1^63^. Overall, the CO algorithm is based on intelligently utilizing these hunting strategies during hunting periods (iterations).

- *Searching*: Cheetahs need to search, including scanning or active search, in their territories (search space) or the surrounding area to find their prey.
- *Sitting-and-waiting*: After the prey is detected, but the situation is not proper, cheetahs may sit and wait for the prey to come nearer or for the position to be better;
- *Attacking*: This strategy has two essential steps:
  - Rushing: When the cheetah decides to attack, they rush toward the prey with maximum speed.
  - Capturing: The cheetah used speed and flexibility to capture the prey by approaching the prey.
- *Leave the prey and go back home*: Two situations are considered for this strategy. (1) If the cheetah is unsuccessful in hunting the prey, it should change its position or return to its territory. (2) In cases with no successful hunting action in some time interval, it may change its position to the last prey detected and searched around it.

The mathematical models of the hunting mentioned above strategies are provided in detail in the following sections. Then, the CO is outlined.

### Search strategy

Cheetahs seek prey in two ways; either scan the environment while sitting or standing or actively patrols the surrounding area. Scanning mode is more suitable when the prey is dense and grazing while walking on the plains. On the other hand, choosing an active mode that needs more energy than the scan mode is better if the prey is scattered and active. Therefore, during the hunting period, depending on the prey's condition, the coverage of the area, and the condition of the cheetahs themselves, a chain of these two search modes may be selected by the cheetah. To mathematically model this searching strategy of cheetahs, let $X_{i,j}^{t}$ denote the current position of cheetah *i* (*i* = 1, 2, …, *n*) in arrangement *j* (*j* = 1, 2, …, *D*), where *n* is the number of cheetahs population and *D* is the dimension of the optimization problem. Indeed, each cheetah experiences different situations dealing with various prey. Each prey is a location of a decision variable corresponding to the best solution while the cheetah's states (other arrangements) construct a population.

**Figure 2:**
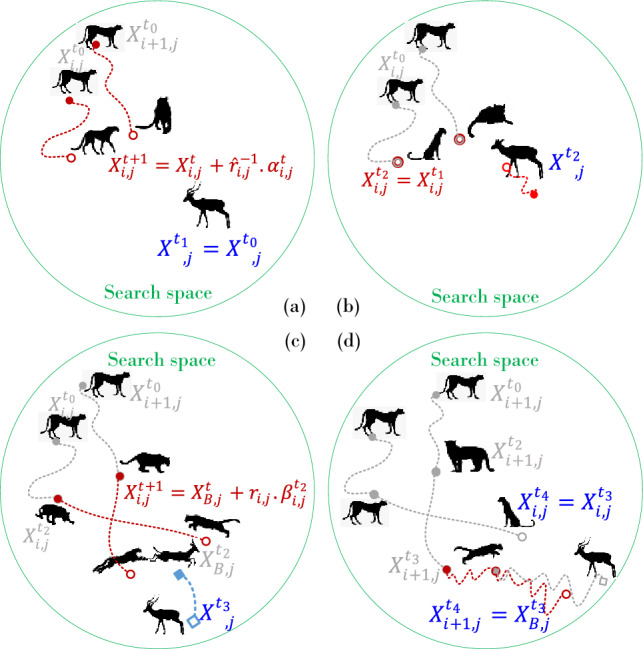
Graphical information of CO's strategies.

Then, the following random search equation is proposed for updating the new position of cheetah *i* in each arrangement based on their current position, and an arbitrary step size as follows:

1 $$X_{i,j}^{t + 1} = X_{i,j}^{t} + \hat{r}_{i,j}^{ - 1} .\alpha_{i,j}^{t}$$

where $X_{i,j}^{t + 1}$ and $X_{i,j}^{t}$ are the next and the current positions of cheetah *i* in arrangement *j*, respectively. Index *t* denotes the current hunting time, and $T$ is the maximum length of hunting time. $\hat{r}_{i,j}^{ - 1}$ and $\alpha_{i,j}^{t}$ are the randomization parameter and step length for cheetah *i* in arrangement *j*, respectively. The second term is the randomization term, where the randomization parameter $\hat{r}_{i,j}^{{}}$ is normally distributed random numbers from a standard normal distribution. The step length $\alpha_{i,j}^{t} > 0$ in most cases can be set at $0.001 \times t/T$ as cheetahs are slow-walking searchers. In encountering other hunters (enemies), the cheetahs may escape rapidly and change their directions. To reflect such behavior as well as near/far destination search mode, the random number $\hat{r}_{i,j}^{ - 1}$ is used here for each cheetah in different hunting periods. In some cases, $\alpha_{i,j}^{t}$ can be adjusted by the distance between the cheetah *i* and his/her neighborhood or leader. The position of a cheetah (named leader) in each arrangement of cheetahs is updated by assuming $\alpha_{i,j}^{t}$ equal to $0.001 \times t/T$ multiplied by the maximum step size (here, we consider it based on the variable limits, i.e., the upper limit minus the lower limit). For other members, $\alpha_{i,j}^{t}$ in each cheetah's arrangement is calculated by multiplying the distance between the position of cheetah *i* and a randomly selected cheetah. Figure 2a illustrates the search strategy.

There is a distance between the leader and the prey (i.e., the best solution in this paper). Thus, the leader position is selected based on the prey position by changing some variables in the best solution. It is to be expected that the leader and the prey would be closer by the time unless the hunting time is terminated, leading to an updated leadership position. It should be noted that the step size of the cheetah is entirely random, and the CO would consider this. Therefore, the CO can effectively solve the optimization problems correctly by utilizing any randomization parameter and random step size, i.e.,${ }\hat{r}_{i,j}^{ - 1}$ and $\alpha_{i,j}^{t}$.

### Sit-and-wait strategy

During the searching mode, the prey may expose to a cheetah's field of vision. In this situation, every movement of the cheetah may make the prey aware of his/her presence and lead to the escape of the prey. To avoid this concern, the cheetah may decide to ambush (by lying on the ground or hiding among the bushes) to get close enough to the prey. Therefore, in this mode, the cheetah remains at his/her position and waits for the prey to come nearer (see Fig. 2b). This behavior can be modeled as follows:

2 $$X_{i,j}^{t + 1} = X_{i,j}^{t}$$

where $X_{i,j}^{t + 1}$ and $X_{i,j}^{t}$ are the updated and current positions of cheetah *i* in arrangement *j*, respectively. This strategy requires the CO algorithm not to change all cheetahs simultaneously in each group to increase the success of hunting (finding a better solution) and hence can assist it in avoiding premature convergence.

### Attack strategy

Cheetahs use two crucial factors to attack their prey: speed and flexibility. When a cheetah decides to attack, he/she rushes to the prey at full speed. After a while, the prey notices the cheetah's attack and begins to flee. The cheetah rapidly pursues the prey in the interception path with its keen eyes, as shown in Fig. 2c. In other words, the cheetah follows the position of the prey and adjusts its direction of movement in such a way as to block the prey's way at one point. Because the cheetah has reached a short distance from the prey at maximum speed, the prey must escape and change its position suddenly to survive, as shown in Fig. 2d, i.e., the next position of the cheetah is near the last position of prey. Also, as shown in Fig. 2d, probably, the one cheetah doesn't participate in an attacking strategy that completely matches the natural hunting of cheetahs. The cheetah uses speed and flexibility to capture the prey in this phase. In a group hunting method, each cheetah may adjust his/her position based on the fleeing prey and the position of the leader or neighborhood cheetah. Simply, these all attacking tactics of cheetahs are mathematically defined as follows:

3 $$X_{i,j}^{t + 1} = X_{B,j}^{t} + \check{r}_{i,j} .\beta_{i,j}^{t}$$

where $X_{B,j}^{t}$ is the current position of the prey in arrangement *j*. In other words, it is the current best position of the population. $\check{r}_{i,j}$ and $\beta_{i,j}^{t}$ are respectively the turning factor and interaction factor associated to the cheetah *i* in arrangement *j*. $X_{B,j}^{t}$ is used in (3) because, in attacking mode, the rushing strategy of cheetahs by utilizing maximum speed helps them get as close as possible to the prey's position in a short time. Hence, this paper calculates the new position of the *i*-th cheetah in attacking mode based on the prey's current position. In the second term, the turning factor $\beta_{i,j}^{t}$ reflects the interaction between the cheetahs or between a cheetah and leader in the capturing mode. Mathematically, this factor can be defined as the difference between the neighborhood cheetah's position, $X_{k,j}^{t}$ ($k \ne i$), and the *i*-th cheetah's position, $X_{i,j}^{t}$. The turning factor $\check{r}_{i,j}$ is a random number that is equal to $|r_{i,j} |^{{{\text{exp}}\left( {r_{i,j} /2} \right)}} {\text{sin}}\left( {2\pi r_{i,j} } \right)$ in this paper. $r_{i,j}$ is normally distributed random numbers from a standard normal distribution. This factor reflects the sharp turns of the cheetahs in the capturing mode.

### Hypotheses

Based on the behaviors of cheetahs in haunting, the following assumptions are considered in the proposed CO algorithm:

1. Each row of the cheetahs’ population is modeled as a cheetah in different states. Each column represents a specific arrangement of cheetahs concerning the prey (best solution of each decision variable). In other words, cheetahs follow their prey (best point of a variable). To find the best optimal solution, the cheetahs require success in capturing the prey in each arrangement. The performance of each cheetah is evaluated by the value of fitness function for that cheetah in all arrangements. The higher performance of a cheetah indicates a higher probability of hunting success.
2. In a real group hunting process, the reactions of all cheetahs are different from others. Indeed, in every arrangement, a cheetah may be in attacking mode while the other cheetahs may be in one of searching, sitting-and-waiting, and attacking modes. Also, the cheetahs' energy is independent of the prey. Modeling each decision variable as an arrangement of cheetahs, besides using the random parameters $\hat{r}_{i,j}^{ - 1}$ and $\check{r}_{i,j}$ results in preventing premature convergence even in an extremely large evolution process. These random variables can be considered an energy source for cheetahs during the hunting process. These two critical ideas have been neglected in previous hunting evolutionary methods that significantly affect optimization performance. In the attacking strategy, the cheetah's direction depends on the prey, but the cheetah movements have completely random behavior in the searching strategy.
3. The behaviors of cheetahs during the searching or the attacking strategies are assumed to be completely random, as depicted in Fig. 2 by the red dash-line, but during the rushing and the capturing mode, the prey scape in a sharp changing direction, as depicted in the last movement shown in Fig. 2d. The randomization parameter $\hat{r}_{i,j}^{{}}$ and the turning factor $\check{r}_{i,j}$ model these random movements. Changing the step length $\alpha_{i,j}^{t}$ and interaction factor $\beta_{i,j}^{t}$ with completely random variables also lead to a suitable optimization process. These confirm that the hunting process is modeled precisely.
4. In the hunting process, the searching or the attacking strategy is deployed randomly, but the searching strategy becomes more likely over time due to decreasing the cheetah's energy level. In some cases, the first steps are devoted to the search strategy, while the attack strategy is selected for large values of *t* to achieve better solutions. Assuming $r_{2}$ and $r_{3}$ as uniformly random numbers from [0, 1]. If $r_{2} \ge r_{3}$ the sit and wait strategy is selected; otherwise, one of the searching or the attacking strategies is selected based on a random value $H = {\text{e}}^{{2\left( {1 - t/T} \right)}} \left( {2r_{1} - 1} \right)$ where $r_{1}$ is a uniformly random number from [0, 1]. By tuning $r_{3}$, the switching rate between the sit-and-wait strategy and two other strategies can be controlled. For instance, based on our experiences, if the objective function is too sensitive to the changes in some decision variables (this can reflect the sensitivity of prey to the movement of the cheetah), the value of $r_{3}$ can be selected as small random numbers. This situation increases the sit-and-wait mode to be chosen by a cheetah, decreasing the rate of changing decision variables. Hence, the success probability of hunting (finding better solutions) is increased. Increasing $t$ in the $H$ function decreases the chance of choosing the attacking strategy by a cheetah due to the energy limitation. Still, this probability is not zero, which get entirely inspired by the cheetah's behavior. To do this, if $H \ge r_{4}$, attack mode is selected else the search mode is implemented. $r_{4}$ is a random number between 0 and 3. Here, higher values of the $r_{4}$ highlights the exploitation phase while decreasing it increases the exploration process.
5. The scanning and sitting-and-waiting strategies have the same meaning in the CO algorithm, indicating that the cheetah (search agent) has no movement during the hunting period.
6. If the leader fails in hunting in some consecutive hunting processing, the position of a randomly chosen cheetah is changed into the last place of success hunting (i.e., the prey position). Maintaining the prey position among a small population in this algorithm strengthens the exploration phase.
7. Each group of cheetahs has a limitation on the hunting time due to their energy limitations. Hence, if a group couldn't be succussed in a hunting period, the current prey is left, and the group comes back to its home range (initial position in this paper) to rest and then start another hunting. Indeed, a group of cheetahs will return home if their energies (which is modeled by hunting time) decrease and the leader's position is constant. In this condition, the position of leader is also updated. As a result of this strategy, it is possible to avoid getting stuck in local optimum solutions.
8. In each iteration, part of members participated in the evolution process.

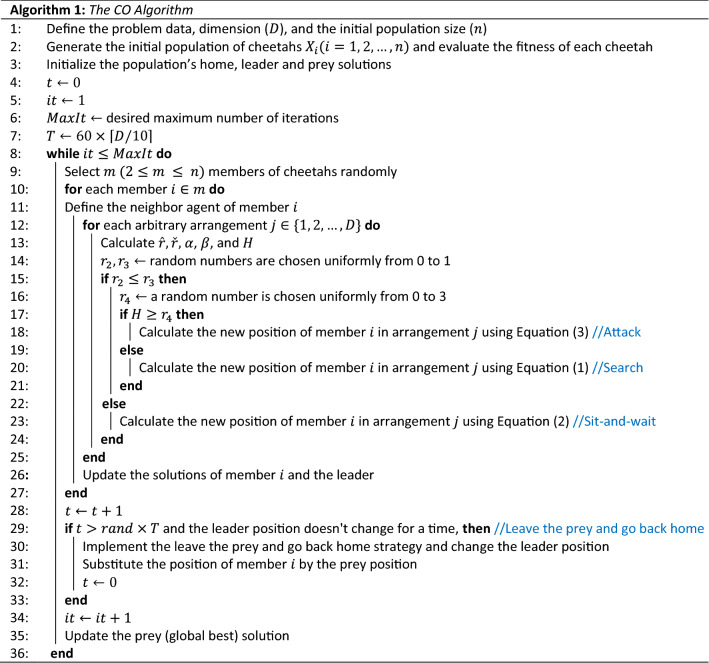
 The essential phases of the CO algorithm may be represented as the pseudo-code summarized in Algorithm 1 based on cheetah hunting techniques and assumptions. The source codes of this algorithm can be found in Supplementary Information.

### Algorithm complexity

When evaluating the performance of an algorithm, its complexity is an important metric. In order to initialize each population in the CO as well as other algorithms such as PSO, GWO, GA, and DE, it requires ${\mathcal{O}}\left( {o \times n} \right)$ time, where $o$ represents the number of objectives and $n$ represents the number of populations. Every algorithm has an ${\mathcal{O}}\left( {MaxIt \times ef} \right)$ complexity, where $MaxIt$ is the maximum number of iterations and $ef$ defines evaluation function complexity for a given problem. If the entire process is simulated, ${\mathcal{O}}\left( N \right)$ would require. Accordingly, algorithms such as PSO, and GWO have a computational complexity of ${\mathcal{O}}\left( {N \times MaxIt \times o \times n \times ef} \right)$. For CO, GA and DE algorithms, the computational complexity is ${\mathcal{O}}\left( {N \times MaxIt \times o \times n \times ef \times \left( {mu + cr} \right)} \right)$, where $mu$ denotes mutation operations, and $cr$ denotes crossover. Table 1 provides information on the average running time of CO and other algorithms for shifted sphere function with 10 variables for 1000 function evaluations and 25 independent runs. The population size for all algorithms is set at 10 and the other parameters of the competitor algorithms are given in Table 2. GWO is more time efficient compared to other approaches in terms of seconds. By contrast, GA exhibit relatively higher computational time than other competitors. The results of Table 1 indicate that, the CO can find better optimal solution in terms of mean and standard deviation (SD) than other algorithms in a reasonable time. It should be noted that the computational time also depends on the coding method of each algorithm. We use typical source codes in this comparison.

**Table 1:** Time comparison of some metaheuristic algorithms performance.

Algorithm	Mean	SD	Average time (s)
CO	4.12	20.01	1.45
PSO	760.37	1212.30	3.16
GA	2942.00	1550.60	4.60
DE	330.97	151.59	1.27
GWO	775.02	662.53	0.75
TLBO	289.37	406.04	1.77

**Table 2:** Parameter setting of competing algorithms for solving 500-D shifted CEC2005 test functions.

Algorithm	Parameter	Value
CO	*N*	6
	*m*	2
DE	*n*	100
	*CR*	0.9
	*F*	0.9
GWO	*n*	40
GA	*n*	50
	*Pc*	0.95
	*Pm*	0.001
PSO	*n*	60
	*w*	0.9
	*c*_*1*_*, c*_*2*_	1.4961
TLBO	*n*	20

## Simulation results

### Results of CO on 500-D shifted CEC2005 test functions

In this benchmark, *f*_1_ to *f*_7_ are classical unimodal functions, and *f*_8_ to *f*_13_ are multimodal functions. This case study is designed to analyze the CO behavior compared to well-known algorithms such as DE, GWO, GA, PSO, and TLBO in large-scale 500-D shifted benchmark functions. In Table 2, the parameters for CO and other competing algorithms are shown. Since the population number differs from one case to another, the maximum number of function evaluations is set to 12 × 10^5^ for all algorithm to have a firm comparison. The statistical results of this case are presented in Table 3. These results confirm the superiority of CO algorithm over the competing algorithms for nine test functions *f*_1_, *f*_3_, *f*_4_, *f*_6_, *f*_7_, *f*_9_, *f*_10_, *f*_12_, and *f*_13_ in terms of mean and standard deviation. For test functions *f*_2_, and *f*_11_, DE algorithm is better than the other algorithms. GA provides better solution for test function *f*_8_, and TLBO shows better solution for test function *f*_5_. Also, according to Freidman’s rank test, the proposed CO algorithm shows the best performance among different competitor algorithms.

**Table 3:** Results of 500-D shifted CEC2005 test functions.

FUN	Properties	CO	DE	GWO	GA	PSO	TLBO
*f*_1_	Mean	**8.46E−04**	1.29E−03	8.31E+05	3.16E+01	5.74E+05	1.37E+06
	SD	**3.90E−05**	2.21E−04	3.55E+04	8.01E+00	4.56E+04	3.66E+04
*f*_*2*_	Mean	4.81E−01	**1.14E−04**	1.55E+03	7.17E+01	1.05E+03	2.99E+03
	SD	1.32E−02	**1.39E−05**	6.31E+01	2.95E+00	7.80E+01	1.34E+02
*f*_*3*_	Mean	**1.60E+05**	8.80E+06	2.73E+06	8.16E+05	5.17E+06	8.56E+06
	SD	**1.04E+04**	4.38E+05	1.98E+05	8.36E+03	7.42E+05	1.47E+06
*f*_*4*_	Mean	**3.44E+01**	7.82E+01	9.37E+01	6.09E+01	1.31E+02	9.97E+01
	SD	**1.88E+00**	3.13E+00	8.16E−01	1.74E+00	7.72E+00	2.07E−01
*f*_*5*_	Mean	1.06E+03	1.51E+04	3.21E+09	2.72E+03	2.74E+09	**4.98E+02**
	SD	2.28E+02	3.13E+03	2.10E+08	3.18E+02	5.35E+08	**1.36E−01**
*f*_*6*_	Mean	**2.51E−01**	2.52E−01	7.88E+05	2.11E+01	5.79E+05	1.29E+06
	SD	**5.45E−05**	2.46E−04	4.68E+04	8.25E+00	3.74E+04	4.71E+04
*f*_*7*_	Mean	**1.09E−01**	1.21E+00	2.29E+04	1.08E+00	2.04E+04	5.28E+04
	SD	**9.30E−03**	1.17E−01	1.14E+03	9.25E−02	4.22E+03	1.42E+03
*f*_*8*_	Mean	−1.71E+05	−1.32E+05	−6.47E+04	**−2.09E+05**	−1.18E+05	−3.13E+04
	SD	3.13E+03	4.81E+03	4.07E+03	**2.45E+02**	4.45E+03	3.90E+03
*f*_*9*_	Mean	**4.20E+01**	4.00E+03	6.06E+03	7.28E+02	6.25E+03	8.16E+03
	SD	**5.21E+00**	6.81E+01	1.73E+02	3.25E+01	3.34E+02	3.69E+02
*f*_10_	Mean	**7.40E−03**	6.48E+00	1.96E+01	7.71E+00	2.04E+01	2.00E+01
	SD	**9.00E−04**	3.31E+00	1.31E−01	3.71E−01	3.77E−01	1.62E−03
*f*_11_	Mean	3.20E−01	**1.59E−02**	6.74E+03	1.13E+00	4.83E+03	1.14E+04
	SD	3.37E−01	**4.95E−02**	4.54E+02	1.48E−01	6.10E+02	3.60E+02
*f*_12_	Mean	**6.22E−04**	1.35E+06	7.35E+09	2.47E−03	7.58E+09	1.45E+10
	SD	**1.97E−03**	4.12E+06	6.13E+08	1.07E−03	3.04E+09	7.27E+08
*f*_13_	Mean	**4.00E−01**	8.00E+04	1.32E+10	1.48E+00	1.23E+10	2.84E+10
	SD	**6.64E−06**	2.81E+04	1.27E+09	2.45E−01	1.63E+09	1.69E+09
Mean rank in		**1.307692**	2.769231	4.615385	2.384615	4.538462	5.384615
Freidman test							

Significant values are in [bold].

One of the major features of CO algorithm is the high capability in the exploitation phase, where the proposed strategies keep the population diversity and search the whole feasible search space, even in large scale optimization problem with small size of initial population, i.e., *n* = 6. Indeed, in all objective functions, the population moves towards better solutions in consecutive iterations without trapping in local optima.

### Results of CO on shifted-rotated CEC2005 test functions

The set of benchmarks with 14 shifted-rotated CEC2005 functions with 30 variables are tested in this section. The results of CO are compared with several powerful, well-known evolutionary algorithms such as whale optimization algorithm (WOA)^44^, emperor penguin optimizer (EPO)^64^, slime mould algorithm (SMA)^65^, Jaya^66^, heat transfer search (HTS)^67^, modified particle swarm optimizer (MPSO)^68^, self-adaptive DE (jDE)^69^, DE^70^, and global and local real-coded genetic algorithms based on parent-centric crossover operators (GL-25)^71^. The parameters of each algorithm are reported in Table 4 and for a fair comparison the number of fitness evaluations are set to 3 × 10^5^ for all algorithms.

**Table 4:** Parameter setting of competing algorithms for solving 30-D shifted-rotated CEC2005 test functions.

Algorithm	Parameter value
CO	*n* = 6, *m* = 2
WOA	*n* = 30, *a1* ∈ [2 0]; *a2* ∈ [− 2 − 1]; *b* = 1
EPO	*n* = 80, *A* ∈ [− 1.5, 1.5], *T*′ ∈ [1, 1000], *S* ∈ [0, 1.5], *M* = 2, *f* ∈ [2, 3], *l* ∈ [1.5, 2]
SMA	*n* = 30, *z* = 0.03, *a* ∈ [− 1, 1], *b* ∈ [0, 1]
Jaya	*n* = 50, *r* ∈ [0, 1]
HTS	*n* = 50, *R* ∈ [0,1]
MPSO	*n* = 40, *w* = 0.9, *c*_1_ = *c*_2_ = 1.4961
GL-25	Not Available
DE	*n* = 100, *CR* = 0.9, *F* = 0.9
jDE	*n* = 60, *F*_l_ = 0.1, *F*_*u*_ = 0.9, *τ*_1_ = *τ*_2_ = 0.1

The performance of CO is analyzed through the searching history, convergence, and trajectory curves. Also, some aspects of CO are cleared using the curves of minimum, maximum and mean value of the objective function in each iteration. Table 5 compares the statistical results of CO with nine evolutionary algorithms. As can be observed from Table 5, the proposed CO algorithm in all functions provides better solution than the WOA, EPO, SMA, Jaya, HTS, DE, and jDE algorithms. Also, in all test functions except *f*_11_, CO algorithm overcomes the MPSO algorithm. Compared to GL-25, CO algorithm has been able to get better solutions for nine functions, i.e., functions *f*_2_, *f*_3_, *f*_7_, *f*_8_, *f*_9_, *f*_11_, *f*_12_, *f*_13_, and *f*_14_. By contrast, for *f*_1_, *f*_4_, *f*_5_, *f*_6_, *f*_10_ GL-25 is better than other algorithms. Mean rank values from Freidman test reveal that CO algorithm has the best performance among the competitor algorithms for solving 30-D shifted-rotated CEC2005 test functions.

**Table 5:** Comparison results between CO and nine evolutionary algorithms on shifted rotated CEC2005 test functions.

Algorithm			CO	WOA	EPO	SMA	Jaya	HTS	MPSO	GL-25	DE	jDE
Unimodal functions	*f*_1_	Mean	5.70E−13	1.22E+03	8.47E+03	1.27E+01	4.86E+03	7.46E+01	6.05E+02	**5.60E−27**	7.69E+03	5.38E−12
		SD	1.76E−12	8.24E+02	5.29E+03	2.80E+01	2.39E+03	3.76E+01	2.24E+02	**1.76E−26**	1.65E+03	2.12E−12
	*f*_*2*_	Mean	**3.25E−05**	5.87E+03	5.17E+03	3.93E+02	2.80E+03	1.49E+02	2.31E+03	4.04E+01	3.93E+04	1.23E+02
		SD	**1.27E−05**	1.95E+04	3.05E+03	1.75E+02	2.27E+03	9.37E+01	4.40E+02	6.28E+01	6.07E+03	2.00E+01
	*f*_*3*_	Mean	**8.02E+05**	1.26E+08	2.29E+08	2.44E+07	4.50E+07	7.24E+07	1.61E+07	2.19E+06	1.04E+08	4.21E+06
		SD	**4.11E+05**	3.45E+08	6.21E+07	7.14E+06	8.32E+06	3.16E+07	1.04E+07	1.08E+06	4.00E+07	9.02E+05
	*f*_*4*_	Mean	1.52E+03	6.27E+04	3.75E+03	1.68E+03	2.52E+04	1.62E+03	1.11E+04	**9.07E+02**	5.05E+04	5.99E+03
		SD	7.71E+02	5.77E+04	5.99E+02	1.90E+03	9.37E+03	1.21E+03	4.01E+03	**4.25E+02**	1.51E+04	1.36E+03
	*f*_*5*_	Mean	5.82E+03	1.01E+04	1.89E+04	9.21E+03	7.54E+03	7.49E+03	6.62E+03	**2.51E+03**	1.13E+04	5.14E+03
		SD	1.31E+03	7.93E+03	9.25E+03	3.16E+03	2.55E+03	3.92E+03	2.25E+03	**1.96E+02**	1.42E+03	7.36E+02
Basic multimodal functions	*f*_*6*_	Mean	5.10E+01	5.25E+08	3.76E+07	1.06E+04	1.21E+04	1.19E+06	2.14E+07	**2.15E+01**	5.89E+08	3.57E+01
		SD	6.61E+01	2.74E+08	2.43E+07	7.87E+03	6.16E+03	9.12E+05	2.38E+07	**1.17E+00**	3.41E+08	3.46E+00
	*f*_*7*_	Mean	**2.06E−02**	3.59E+02	5.36E+02	5.47E+01	3.85E+00	3.76E+02	1.37E+02	2.78E−02	4.70E+03	6.22E−02
		SD	**1.71E−02**	2.60E+01	4.29E+02	1.25E+01	1.43E+00	3.02E+02	7.92E+01	3.62E−02	2.73E+00	1.97E−02
	*f*_*8*_	Mean	**2.02E+01**	2.13E+01	2.15E+01	2.11E+01	2.12E+01	2.13E+01	2.07E+01	2.09E+01	2.11E+01	2.09E+01
		SD	**7.71E−02**	1.15E−01	1.26E+00	9.93E−0	7.39E−01	9.17E−02	1.03E−01	5.94E−02	2.86E−02	4.59E−02
	*f*_*9*_	Mean	**2.03E−10**	4.00E+02	5.34E+02	2.27E+02	3.02E+02	2.07E+02	1.65E+02	2.45E+01	2.66E+02	4.34E+01
		SD	**8.37E−10**	8.52E+01	4.29E+01	6.18E+01	9.38E+01	7.99E+01	3.73E+01	7.35E+00	2.48E+01	6.04E+00
	*f*_10_	Mean	1.99E+02	6.16E+02	3.48E+02	5.39E+02	5.05E+02	4.39E+02	2.07E+02	**1.42E+02**	3.37E+02	1.92E+02
		SD	5.53E+01	7.20E+01	7.90E+01	2.04E+02	3.14E+02	2.72E+02	6.82E+01	**6.45E+01**	1.24E+01	1.35E+01
	*f*_11_	Mean	2.69E+01	4.18E+01	6.14E+01	4.26E+01	5.96E+01	6.80E+01	**2.68E+01**	3.27E+01	4.27E+01	2.79E+01
		SD	3.26E+00	2.92E+01	1.19E+01	3.33E+01	4.47E+01	5.64E+01	**4.03E+00**	7.79E+00	7.98E−01	1.18E+00
	*f*_12_	Mean	**1.76E+03**	2.35E+05	3.59E+05	1.29E+04	1.70E+05	6.00E+04	8.92E+04	6.53E+04	4.84E+05	3.78E+04
		SD	**2.25E+03**	5.79E+03	9.37E+04	8.14E+03	7.31E+04	3.18E+04	3.90E+04	4.69E+04	1.27E+05	5.63E+03
Expanded multimodal functions	*f*_13_	Mean	**1.32E+00**	1.93E+01	1.70E+01	1.90E+01	1.87E+01	1.67E+01	1.08E+01	6.23E+00	2.47E+01	6.04E+00
		SD	**3.04E−01**	2.28E+00	1.60E+00	2.04E+00	1.04E+00	2.85E+00	1.64E+00	4.88E+00	1.50E+00	5.82E−01
	*f*_14_	Mean	**1.28E+01**	1.49E+01	1.55E+01	1.60E+01	1.40E+01	1.38E+01	1.32E+01	1.31E+01	1.39E+01	1.29E+01
		SD	**3.32E−01**	8.49E−01	1.10E+01	7.19E−01	5.38E−01	6.47E−01	1.05E−01	1.84E−01	1.90E−01	1.56E−01
Mean rank in Freidman test			1.642857	8.321429	8.5	5.75	6.857143	5.892857	4.642857	2.25	8.25	2.892857

Significant values are in [bold].

**Figure 3:**
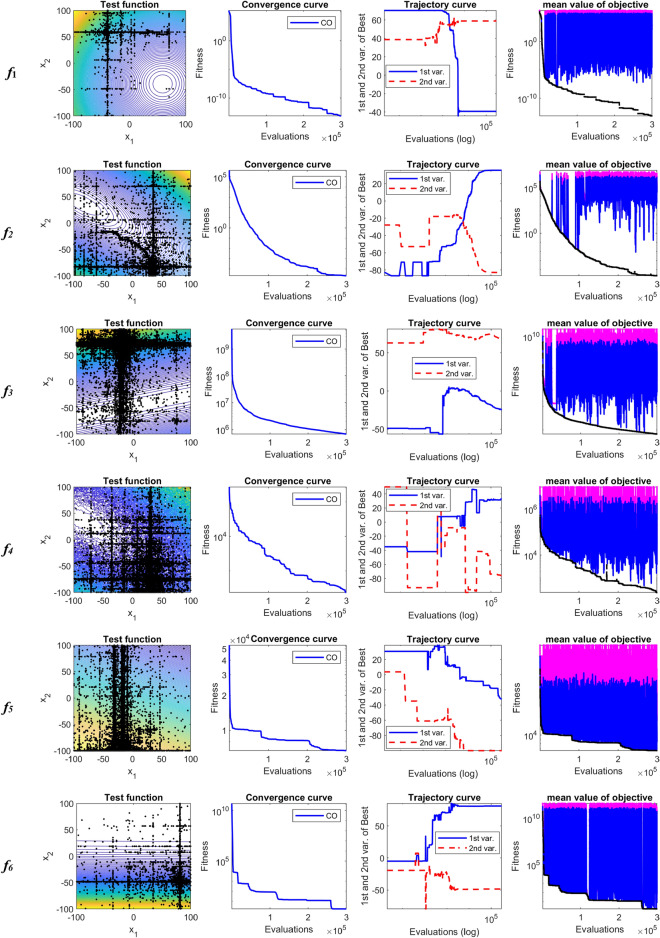

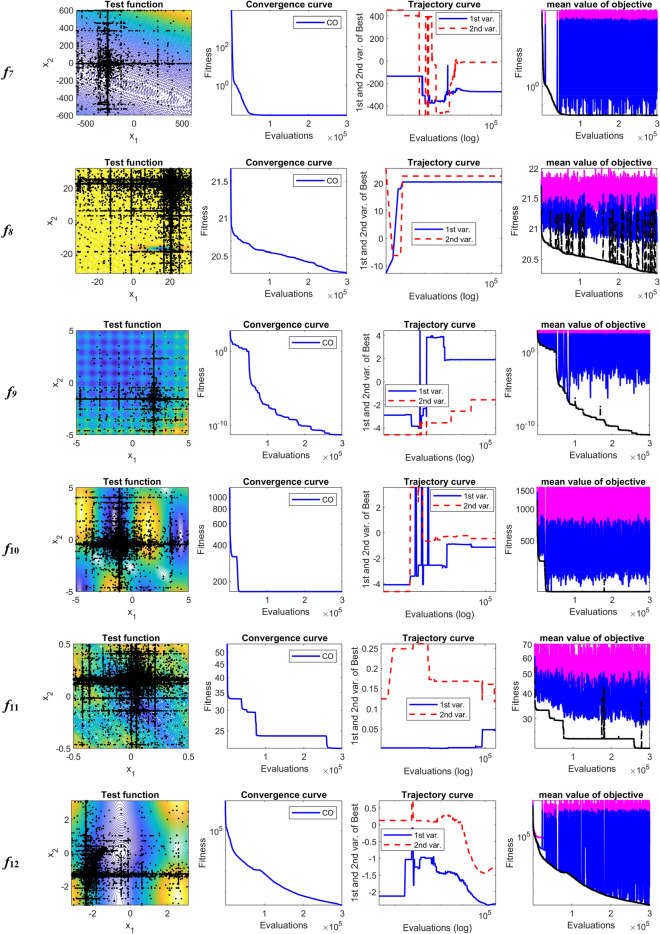

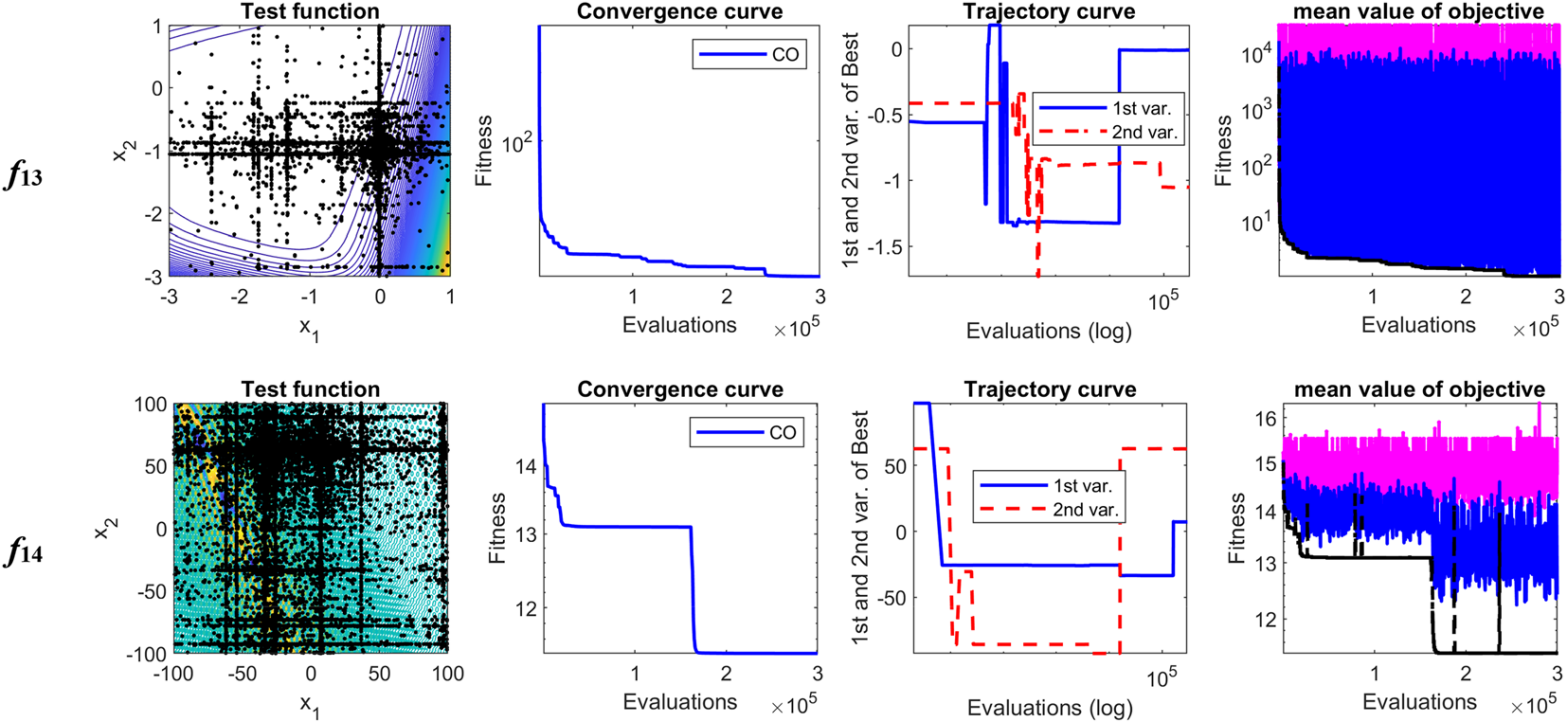
Convergence characteristics of CO on shifted-rotated CEC2005 benchmark functions.

The plotted search history in Fig. 3 shows that the CO gains a better solution through exploring the feasible search space. As shown in Fig. 3, each variable changes its location from the lower limit to its upper limits several times, which guarantees to find suitable solutions. This regular searching history is a unique feature in the CO. The first graph from the right shows the minimum, maximum and average values of the objective function corresponding to the population in each iteration. Also, Fig. 3 shows that the leave the prey and go back home strategy keeps the population diversity during the evolution process. The cheetah's strategies are adopted to proceed with the optimal solution despite population diversity in each step of evolution.

### Results of CO on large scale optimization problems

In this section, to investigate how the CO method deals with the large-scale optimization problem, it is tested on CEC-2010 and CEC-2013 large-scale functions to validate its performance. The results of CO in the original condition are compared to other well-known modified algorithms which are tuned to solve the large-scale optimization problems, including^72^: (1) SLPSO, (2) CSO, (3) DMS-L-PSO, (4) CCPSO2, (5) DECC-G, (6) MLCC, and (7) DECC-DG methods as presented in Table 6. The results show that the CO can effectively enhance the results of eight benchmark functions while revealing completely comparable results in other objective functions. In the case of large-scale optimization problems, the CO uses 6-population while 2 of them are called in each iteration. The maximum number of function evaluations for all algorithms are set to 3 × 10^6^ for these problems.

**Table 6:** Comparison of results of CO and state-of-the-art methods on the CEC2010 large-scale functions.

FUN	Properties	CO	CSO	CCPSO2	DECC-DG	MLCC	DMS-L-PSO	DECC-G	SLPSO
*f*_*1*_	Mean	2.4E−3	4.5E−12	1.6E+0	16.1E+0	**000.0E+0**	4.9E+9	13.6E−15	8.7E−18
	SD	179.0E−6	778.0E−15	1.7E+0	11.9E+0	**000.0E+0**	231.0E+6	1.7E−15	519.0E−21
*f*_*2*_	Mean	115.3E+0	7.4E+3	7.5E+0	4.5E+3	**332.0E−3**	6.2E+3	48.8E+0	1.9E+3
	SD	12.6E+0	286.0E+0	1.7E+0	209.0E+0	**543.0E−3**	187.0E+0	13.1E+0	112.0E+0
*f*_*3*_	Mean	14.1E−6	**2.6E−9**	8.9E−3	16.7E+0	81.7E−3	17.4E+0	1.7E+0	1.9E+0
	SD	273.0E−9	**452.0E−12**	11.7E−3	271.0E−3	311.0E−3	56.1E−3	288.0E−3	166.0E−3
*f*_*4*_	Mean	**107.0E+9**	725.0E+9	1.2E+12	3.8E+12	15.4E+12	39.0E+12	12.5E+12	299.0E+9
	SD	**23.0E+9**	123.0E+9	741.0E+9	675.0E+9	6.6E+12	4.4E+12	2.9E+12	71.6E+9
*f*_*5*_	Mean	279.0E+6	**11.5E+6**	453.0E+6	154.0E+6	313.0E+6	104.0E+6	260.0E+6	31.7E+6
	SD	61.6E+6	**1.6E+6**	118.0E+6	19.0E+6	109.0E+6	7.8E+6	81.6E+6	6.2E+6
*f*_*6*_	Mean	8.5E+6	**821.0E−9**	19.2E+6	16.4E+0	16.1E+6	1.7E+6	4.8E+6	20.8E+0
	SD	1.8E+6	**46.1E−9**	1.7E+6	273.0E−3	4.4E+6	442.0E+3	614.0E+3	4.0E+0
*f*_*7*_	Mean	**92.8E−6**	20.1E+3	170.0E+6	5.8E+3	1.8E+6	24.2E+9	8.4E+6	64.9E+3
	SD	**15.0E−6**	3.9E+3	323.0E+6	2.7E+3	2.9E+6	1.6E+9	7.6E+6	56.0E+3
*f*_*8*_	Mean	**4.2E+6**	38.7E+6	33.1E+6	39.4E+6	37.6E+6	143.0E+6	47.2E+6	7.8E+6
	SD	**11.4E+6**	68.1E+3	29.7E+6	29.8E+6	32.7E+6	34.2E+6	30.8E+6	1.6E+6
*f*_*9*_	Mean	**25.2E+6**	70.3E+6	114.0E+6	59.5E+6	119.0E+6	5.8E+9	254.0E+6	33.0E+6
	SD	**2.2E+6**	5.7E+6	36.0E+6	9.2E+6	14.4E+6	202.0E+6	10.1E+6	4.5E+6
*f*_*10*_	Mean	4.9E+3	9.6E+3	5.7E+3	4.6E+3	3.0E+3	5.9E+3	9.2E+3	**2.6E+3**
	SD	175.0E+0	76.7E+0	1.0E+3	121.0E+0	370.0E+0	248.0E+0	437.0E+0	**217.0E+0**
*f*_*11*_	Mean	194.0E+0	**40.2E−9**	198.0E+0	11.3E+0	196.0E+0	182.0E+0	25.2E+0	23.2E+0
	SD	1.5E+0	**6.9E−9**	239.0E−3	504.0E−3	3.1E+0	8.6E+0	1.1E+0	2.1E+0
*f*_*12*_	Mean	**133.0E+0**	437.0E+3	27.8E+3	2.5E+3	36.0E+3	2.8E+6	39.1E+3	17.5E+3
	SD	**15.0E+0**	62.2E+3	7.6E+3	314.0E+0	6.5E+3	110.0E+3	5.8E+3	9.1E+3
*f*_*13*_	Mean	1.2E+3	**629.0E+0**	1.3E+3	4.9E+3	2.4E+3	96.8E+6	3.1E+3	959.0E+0
	SD	610.0E+0	**232.0E+0**	182.0E+0	2.7E+3	1.6E+3	26.2E+6	1.2E+3	374.0E+0
*f*_*14*_	Mean	**78.7E+6**	249.0E+6	322.0E+6	340.0E+6	324.0E+6	5.0E+9	577.0E+6	84.1E+6
	SD	**5.8E+6**	15.3E+6	146.0E+6	18.5E+6	19.7E+6	343.0E+6	21.8E+6	6.3E+6
*f*_*15*_	Mean	9.7E+3	10.1E+3	10.2E+3	**5.8E+3**	7.2E+3	6.2E+3	9.8E+3	11.2E+3
	SD	446.0E+0	52.3E+0	890.0E+0	**60.2E+0**	1.1E+3	276.0E+0	2.7E+3	86.5E+0
*f*_*16*_	Mean	391.0E+0	58.9E−9	397.0E+0	**723.0E−15**	381.0E+0	339.0E+0	85.0E+0	25.1E+0
	SD	1.6E+0	6.3E−9	464.0E−3	**48.9E−15**	47.7E+0	788.0E−3	10.4E+0	2.4E+0
*f*_*17*_	Mean	**3.7E+3**	2.2E+6	141.0E+3	41.8E+3	156.0E+3	2.7E+6	163.0E+3	90.0E+3
	SD	**286.0E+0**	155.0E+3	58.1E+3	1.1E+3	10.3E+3	154.0E+3	9.6E+3	15.8E+3
*f*_*18*_	Mean	2.6E+3	**1.7E+3**	2.9E+3	15.1E+9	6.8E+3	2.8E+9	9.0E+3	2.8E+3
	SD	761.0E+0	**522.0E+0**	373.0E+0	1.9E+9	6.0E+3	530.0E+6	1.1E+3	833.0E+0
*f*_*19*_	Mean	**533.0E+3**	10.1E+6	1.4E+6	1.7E+6	1.3E+6	16.3E+6	733.0E+3	5.1E+6
	SD	**21.2E+3**	564.0E+3	89.0E+3	104.0E+3	105.0E+3	670.0E+3	46.1E+3	705.0E+3
*f*_*20*_	Mean	1.6E+3	**1.1E+3**	2.0E+3	61.7E+9	2.0E+3	4.1E+9	3.5E+3	1.9E+3
	SD	142.0E+0	**159.0E+0**	208.0E+0	5.8E+9	196.0E+0	634.0E+6	245.0E+0	180.0E+0
+ (CO is less)			11	17	15	14	15	13	12
– (CO is greater)			**9**	1	5	5	5	6	8
= (is equal)			**0**	2	0	1	0	1	0

Significant values are in [bold].

A comparison study between CO algorithm with a new modified version of PSO named dynamic group learning distributed particle swarm optimization (DGLDPSO)^73^ is done in Table 7. As can be seen, the CO algorithm can defeat DGLDPSO in 10 functions while almost other objectives reach comparable values. It is worth noting that the CO can also be modified to achieve better solutions.

**Table 7:** Results of CO and DGLDPSO on the CEC2010 large-scale functions.

FUN	Properties	CO	DGLDPSO	FUN	Properties	CO	DGLDPSO
*f*_*1*_	Mean	2.4E−3	**4.55E−21**	*f*_*11*_	Mean	1.94E+2	**3.28E−13**
	SD	1.79E−04	**2.61E−22**		SD	1.52E+00	**1.23E−14**
*f*_*2*_	Mean	**1.153E+2**	7.35E+02	*f*_*12*_	Mean	**1.33E+2**	6.25E+04
	SD	**1.26E+1**	4.52E+1		SD	**1.5E+1**	3.55E+03
*f*_*3*_	Mean	1.41E−05	**2.33E−13**	*f*_*13*_	Mean	1.20E+03	**5.70E+02**
	SD	2.73E−07	**1.55E−14**		SD	6.10E+2	**1.83E+02**
*f*_*4*_	Mean	**1.07E+11**	3.26E+11	*f*_*14*_	Mean	**7.87E+07**	1.46E+08
	SD	**2.30E+10**	5.98E+10		SD	**5.84E+06**	8.59E+06
*f*_*5*_	Mean	2.79E+08	**2.83E+07**	*f*_*15*_	Mean	**9.73E+03**	1.05E+04
	SD	6.16E+07	**1.06E+06**		SD	**4.46E+2**	9.96E+01
*f*_*6*_	Mean	8.51E+06	**4.14E−09**	*f*_*16*_	Mean	3.91E+2	**3.58E−13**
	SD	1.75E+06	**1.39E−10**		SD	1.57E+00	**1.14E−14**
*f*_*7*_	Mean	**9.28E−05**	2.43E+01	*f*_*17*_	Mean	**3.71E+03**	1.84E+06
	SD	**1.50E−05**	1.00E+01		SD	**2.86E+2**	3.03E+05
*f*_*8*_	Mean	**4.18E+06**	2.84E+07	*f*_*18*_	Mean	2.578E+3	**1.97E+03**
	SD	**1.14E+07**	1.99E+5		SD	7.61E+2	**6.27E+02**
*f*_*9*_	Mean	**2.52E+07**	4.47E+07	*f*_*19*_	Mean	**5.33E+5**	5.66E+06
	SD	**2.16E+06**	3.44E+06		SD	**2.12E+4**	2.88E+05
*f*_*10*_	Mean	4.86E+03	**2.05E+03**	*f*_*20*_	Mean	1.563E+3	**1.25E+03**
	SD	1.75E+2	**1.26E+02**		SD	1.42E+2	**1.18E+02**

Significant values are in [bold].

The CEC2013 large-scale benchmark functions^74^ are selected as another case study. Implementing the CO algorithm on these benchmarks is compared with seven well-known modified optimization algorithms adopted to solve the large-scale optimization problems in Table 8. The CO algorithm enhances the results of ten benchmark functions which confirm the effectiveness of CO algorithm in dealing with large-scale optimization problems. The initial populations are set to 6, while 2-population is used in each iteration. Indeed, by changing the turning factor and interaction factor ($\check{r}_{i,j}$ and $\beta_{i,j}^{t}$) and randomization parameter and step length ($\hat{r}_{i,j}^{ - 1}$ and $\alpha_{i,j}^{t}$) the results of other benchmark functions can be enhanced.

**Table 8:** Comparison of results of CO and state-of-the-art methods on the CEC2013 large-scale functions.

FUN	Properties	CO	CSO	CCPSO2	DECC-DG	MLCC	DMS-L-PSO	DECC-G	SLPSO
*f*_*1*_	Mean	111.0E−6	3.7E−12	5.3E+0	1.3E+3	**81.0E−27**	5.7E+9	2.2E−12	10.9E−18
	SD	15.5E−6	1.2E−12	1.3E+0	4.0E+3	**311.0E−27**	144.0E+6	883.0E−15	2.6E−18
*f*_*2*_	Mean	198.0E+0	7.0E+3	12.4E+0	12.8E+3	**8.3E+0**	12.4E+3	49.0E+0	2.1E+3
	SD	24.3E+0	356.0E+0	1.0E+0	462.0E+0	**5.6E+0**	269.0E+0	22.5E+0	136.0E+0
*f*_*3*_	Mean	**20.0E+0**	21.6E+0	20.0E+0	21.4E+0	20.0E+0	21.4E+0	20.1E+0	21.6E+0
	SD	**3.8E−9**	5.4E−3	40.0E−6	15.2E−3	1.7E−3	19.2E−3	2.3E−3	14.5E−3
*f*_*4*_	Mean	**659.0E+6**	12.6E+9	16.1E+9	52.4E+9	87.6E+9	900.0E+9	142.0E+9	4.4E+9
	SD	**277.0E+6**	1.9E+9	7.6E+9	33.6E+9	28.5E+9	37.8E+9	64.9E+9	948.0E+6
*f*_*5*_	Mean	21.8E+6	862.0E+3	17.0E+6	5.8E+6	10.2E+6	5.5E+6	7.5E+6	**841.0E+3**
	SD	3.0E+6	21.3E+3	4.1E+6	349.0E+3	1.9E+6	419.0E+3	1.3E+6	**123.0E+3**
*f*_*6*_	Mean	**1.0E+6**	1.1E+6	1.1E+6	1.1E+6	1.1E+6	1.0E+6	1.1E+6	1.1E+6
	SD	**19.1E+3**	1.1E+3	9.5E+3	904.0E+0	3.7E+3	4.0E+3	582.0E+0	1.5E+3
*f*_*7*_	Mean	**61.9E+3**	7.6E+6	116.0E+6	835.0E+6	430.0E+6	3.6E+9	398.0E+6	1.6E+6
	SD	**15.0E+3**	1.4E+6	87.1E+6	766.0E+6	218.0E+6	261.0E+6	303.0E+6	705.0E+3
*f*_*8*_	Mean	**25.4E+12**	350.0E+12	620.0E+12	4.6E+15	4.6E+15	6.8E+15	2.9E+15	103.0E+12
	SD	**16.5E+12**	35.9E+12	584.0E+12	525.0E+12	3.7E+15	1.4E+15	1.3E+15	36.2E+12
*f*_*9*_	Mean	1.7E+9	**39.4E+6**	3.2E+9	500.0E+6	898.0E+6	505.0E+6	597.0E+6	82.5E+6
	SD	212.0E+6	**6.4E+6**	600.0E+6	22.0E+6	197.0E+6	26.6E+6	119.0E+6	10.6E+6
*f*_*10*_	Mean	92.4E+6	94.1E+6	93.7E+6	94.6E+6	**92.2E+6**	93.1E+6	93.0E+6	92.5E+6
	SD	630.0E+3	149.0E+3	440.0E+3	34.7E+3	**384.0E+3**	311.0E+3	553.0E+3	1.7E+6
*f*_*11*_	Mean	**14.2E+6**	358.0E+9	930.0E+9	23.5E+9	120.0E+9	496.0E+9	59.0E+9	933.0E+9
	SD	**8.9E+6**	9.8E+9	9.6E+9	13.2E+9	27.7E+9	40.1E+9	44.9E+9	9.0E+9
*f*_*12*_	Mean	1.7E+3	**1.3E+3**	2.0E+3	163.0E+9	2.1E+3	4.4E+9	3.4E+3	1.8E+3
	SD	255.0E+0	**82.3E+0**	88.7E+0	16.1E+9	199.0E+0	834.0E+6	269.0E+0	174.0E+0
*f*_*13*_	Mean	**3.9E+6**	806.0E+6	2.0E+9	19.8E+9	8.2E+9	116.0E+9	4.5E+9	465.0E+6
	SD	**1.2E+6**	102.0E+6	554.0E+6	6.1E+9	2.7E+9	10.5E+9	647.0E+6	317.0E+6
*f*_*14*_	Mean	**17.9E+6**	5.2E+9	142.0E+9	18.6E+9	118.0E+9	1.3E+12	75.3E+9	328.0E+6
	SD	**2.0E+6**	2.9E+9	98.7E+9	9.4E+9	68.6E+9	159.0E+9	34.4E+9	517.0E+6
*f*_*15*_	Mean	**775.0E+3**	17.4E+6	3.7E+6	9.5E+6	6.7E+6	1.6E+9	4.8E+6	78.7E+6
	SD	**30.6E+3**	653.0E+3	1.7E+6	984.0E+3	917.0E+3	618.0E+6	421.0E+3	8.5E+6
+ (CO is less)			11	17	13	9	13	12	12
− (CO is greater)			4	2	2	5	2	3	3
= (is equal)			0	1	0	1	0	0	0

Significant values are in [bold].

### Results of CO on the real-life engineering application

The ELD problem is one of the most significant, well-known, and complicated optimization problems in power systems. This problem tries to determine the optimal output power generation of thermal units to meet the power system's required load demand while minimizing the units' fuel expenditures. ELD may also evaluate transmission system power losses and multi-fuel and valve point effects. The primary limitations of this problem include the restriction on load balance, restriction on power generation, restriction on the ramp rate, and restriction on prohibited operation zone.

The detailed ELD formulation is explained in^75^. a 15-unit test system is considered to evaluate the performance of CO algorithm to solve this nonconvex optimization issue. This system's total demand is 2630 MW. Other information about this system can be found in^75^. The results of statistical comparisons between CO and different meta-heuristic and improved algorithms in recent studies are summarized in Table 9. The competitive modified and hybrid algorithms in these two cases are: bacterial foraging optimization (BFO)^76^, a modified ion motion optimization (MIMO) conglomerated with crisscross search (CSO) named C-MIMO-CSO^77^, TLBO^78^, an improved orthogonal design particle swarm optimization (IODPSO) algorithm^79^, synergic predator–prey optimization (SPPO) algorithm^80^, multi-strategy ensemble biogeography-based optimization (MsEBBO)^81^, a new variant for the firefly algorithm, considering a non-homogeneous population named NhFA-Rnp^82^, clustering cuckoo search optimization (CCSO)^83^, a novel variant of competitive swarm optimizer (CSO) referred to as OLCSO^84^, and adaptive charged system search (ACSS)^85^. The results demonstrate that the algorithm outperforms other state-of-the-art and improved algorithms in terms of worst, mean, best, and standard deviation values.

**Table 9:** Statistical results of CO and other metaheuristic methods in solving ELD of the 15-unit test system.

Method	Worst	Mean	Best	SD
BFO^76^	32,784.502	32,976.8120	–	8.58E+01
C-MIMO-CSO^77^	32,701.210	32,701.2101	32,701.2200	5.80E−03
TLBO^78^	32,697.215	32,697.2151	32,697.2151	0.00E+00
IODPSO-L^79^	32,692.390	32,692.3958	32,692.3900	0.00E+00
SPPO^80^	32,708.000	32,732.0866	32,789.0000	1.80E+01
MsEBBO^81^	32,692.397	32,692.3973	32,692.3975	6.09E+05
NhFA-Rnp^82^	32,697.910	32,700.5600	32,709.9400	2.64E+00
CCSO^83^	32,706.640	32,706.6422	32,706.6400	7.00E−04
OLCSO^84^	32,692.3961	32,692.3981	32,692.4033	2.20E−03
ACSS^85^	32,761.3126	32,727.6967	32,678.1290	2.55E+01
CO	**32,678.1866**	**32,678.1465**	**32,678.0999**	**2.75E−02**

Significant values are in [bold].

## Conclusion

We proposed in this paper an optimization algorithm named cheetah optimizer (CO) based on the hunting process of cheetahs in nature. The proposed algorithm relies on several hunting strategies used by cheetahs instead of using mathematically complex approaches. In this regard, each decision variable is considered a possible arrangement of a group of cheetahs. Hence, each population can be regarded as a probable arrangement of cheetahs. The search, sit-and-wait and attack were mathematically modeled as the primary strategies of the proposed CO algorithm. Leave the prey and back to the home strategy was also implemented to enhance the algorithm's abilities in avoiding premature convergence and local optimal entrapment. These concepts were modeled in the CO framework so that it became an easy, fast, and powerful evolutionary method. The experimental results confirmed the monotonic behavior of the CO algorithm in dealing with low- and large-scale optimization problems. Finally, we validated the performance of the CO algorithm over the practical nonconvex ELD problem. The results showed that the suggested algorithm outperformed existing state-of-the-art algorithms in solving complex and challenging optimization problems. The main direction for future works is the development of the multi-objective CO, the application of CO to some complex engineering problems, and the hybridization of the proposed hunting strategies with other evolutionary methods.

## Supplementary Information

Supplementary Information.

## Data Availability

Data sharing is not applicable—no new data is generated.
